# Entry Efficiency, Protease Dependence, and Antibody-Mediated Neutralization of SARS-CoV-2 Sublineages KP.3.1.1 and XEC

**DOI:** 10.3390/vaccines13040385

**Published:** 2025-04-03

**Authors:** Prerna Arora, Amy Kempf, Inga Nehlmeier, Sebastian R. Schulz, Hans-Martin Jäck, Markus Hoffmann, Stefan Pöhlmann

**Affiliations:** 1 Infection Biology Unit, German Primate Center—Leibniz Institute for Primate Research, 37077 Göttingen, Germany; akempf@dpz.eu (A.K.); inehlmeier@dpz.eu (I.N.); mhoffmann@dpz.eu (M.H.); 2 Faculty of Biology and Psychology, Georg-August-University Göttingen, 37073 Göttingen, Germany; 3 Division of Molecular Immunology, Department of Internal Medicine 3, Friedrich-Alexander University of Erlangen-Nürnberg, 91054 Erlangen, Germany; sebastian.schulz@uk-erlangen.de (S.R.S.); hans-martin.jaeck@fau.de (H.-M.J.)

**Keywords:** SARS-CoV-2 variants, BA.2.86 descendants, cell tropism, monoclonal antibody susceptibility

## Abstract

Background: The SARS-CoV-2 variants KP.3.1.1 and XEC currently dominate the COVID-19 epidemic. However, their cell tropism, proteolytic processing, and susceptibility to neutralization by monoclonal antibodies remain incompletely characterized. Methods: We employed pseudotyped viruses to assess the entry efficiency of KP.3.1.1 and XEC in various cell lines, their dependence on TMPRSS2 for lung cell entry, and their ability to use ACE2 for infection. Additionally, we evaluated their susceptibility to neutralization by monoclonal antibodies BD55-4637 and BD55-5514. Results: KP.3.1.1 and XEC entered cell lines with similar efficiency as the parental JN.1 lineage and utilized TMPRSS2 for Calu-3 lung cell entry. Unlike JN.1, KP.3.1.1 and XEC failed to efficiently use murine ACE2 for cell entry. Both variants were effectively neutralized by the monoclonal antibodies BD55-4637 and BD55-5514, suggesting therapeutic potential. Conclusions: Our findings demonstrate that JN.1, KP.3.1.1, and XEC, like their predecessor BA.2.86, rely on TMPRSS2 for lung cell entry and remain sensitive to certain neutralizing monoclonal antibodies. However, these variants differ in their ability to utilize ACE2 species orthologs for cell entry.

## 1. Introduction

The COVID-19 pandemic and subsequent endemic entailed the constant emergence of new SARS-CoV-2 variants with enhanced transmissibility and/or antibody evasion [1,2,3]. Mutations governing transmissibility and immune evasion are mainly located in the viral surface protein spike (S), which facilitates viral entry into host cells and is the main target of neutralizing antibodies [4]. For cell entry, the S protein binds to the receptor ACE2 and employs either the cellular serine protease TMPRSS2, which is located at the plasma membrane, or the endo/lysosomal cysteine protease cathepsin L for S protein activation by cleavage [5,6,7]. Spike mutations can alter ACE2 usage and the choice of activating protease [8,9]. Additionally, mutations in the N-terminal domain (NTD) and, particularly, the receptor-binding domain (RBD) of the S protein can confer resistance to neutralizing antibodies induced by vaccination or infection or employed for COVID-19 therapy [1,10,11].

The highly mutated BA.2-derived variant BA.2.86 emerged in 2023 and constitutes the first Omicron sublineage that enters Calu-3 lung cells in a TMPRSS2-dependent fashion and with the same efficiency as the virus that started the pandemic in Wuhan, China, in 2019 [12,13,14,15]. BA.2.86 showed slightly increased sensitivity to antibody-mediated neutralization as compared to contemporary variants [12,16] and did not become globally dominant. However, the BA.2.86 descendant JN.1 attained global dominance, likely due to amino acid exchange L455S in the S protein, which reduced sensitivity to antibody-mediated neutralization [17,18,19,20]. At, present, the JN.1 sublineages KP.3.1.1 and XEC, a recombinant variant derived from KS.1.1 and KP.3.3, exhibit high prevalence at the global level [21,22], likely due to increased antibody evasion. Thus, deletion of S31 in the NTD of KP.3.1.1 S protein enhances antibody evasion through glycosylation-mediated shielding [23], and T22N and F59S in the XEC S protein modify hydrophobic interactions affecting conformational stability of the S protein and neutralization sensitivity [24]. However, only limited information is available on the impact of mutations in the KP.3.1.1 and XEC S proteins on cell tropism, S protein cleavage, and activation and sensitivity to monoclonal antibodies.

Here, employing pseudotyped viruses, we report that JN.1, KP.3.1.1, and XEC, like BA.2.86, depend on TMPRSS2 activity for lung cell entry and are sensitive to neutralization by certain monoclonal antibodies while only JN.1 but not KP.3.1.1 and XEC can employ murine ACE2 for robust cell entry.

## 2. Materials and Methods

### 2.1. Cell Lines and Culture Conditions

The following cell lines that were used for this study were incubated at 37 °C in a humidified atmosphere with 5% CO_2_:


**Cell Line**

**Species and Organ**

**Source (Catalogue No. and RRID)**

**Culture Medium and Conditions**

**Special Characteristics/Method**
BHK-21Syrian hamster, kidneyATCC, Cat# CCL-10, RRID: CVCL_1915Dulbecco’s Modified Eagle Medium (DMEM; PAN-Biotech, Aidenbach, Germany) with 10% FCS and 1% penicillin–streptomycin (P/S) at 37 °C, 5% CO_2_Transfected with Lipofectamine 2000 (Thermo Fisher Scientific, Waltham, MA, USA), following the manufacturer’s instructionsVeroAfrican green monkey, kidney, femaleATCC, Cat# CRL-1586, RRID: CVCL_0574DMEM (PAN-Biotech, Aidenbach, Germany) with 10% FCS and 1% P/S at 37 °C, 5% CO_2_
293THuman, kidney, femaleDSMZ, Cat# ACC-635, RRID: CVCL_0063DMEM (PAN-Biotech, Aidenbach, Germany) with 10% FCS and 1% P/S at 37 °C, 5% CO_2_Transfected via calcium phosphate precipitation methodCaco-2Human, colon, maleATCC, Cat# HTB-37, RRID: 0025DMEM (PAN-Biotech, Aidenbach, Germany) with 10% FCS and 1% P/S at 37 °C, 5% CO_2_
Calu-3Human, lung, maleATCC, Cat# HTB-55, RRID: CVCL_0609DMEM (PAN-Biotech, Aidenbach, Germany) with 10% FCS and 1% P/S at 37 °C, 5% CO_2_
Calu-3-Omega Human, lung, male
DMEM/F-12 (Thermo Fisher Scientific, Waltham, MA, USA) with 10% FCS, 1% P/S, NEAA, sodium pyruvate, and 2 µg/mL puromycin at 37 °C, 5% CO_2_Expressing beta-galactosidase omega fragment, generated by retroviral transduction and puromycin selection [25]293T ACE2 Knockout (293T_ACE2 KO)Human, kidney, female
DMEM (PAN-Biotech, Aidenbach, Germany) with 10% FCS and 1% P/S at 37 °C, 5% CO_2_Generated via CRISPR-Cas9 system, maintained with 1 µg/mL puromycin [26,27]

The cell lines were regularly controlled for mycoplasma contamination, and cell line authentication was performed using short tandem repeat (STR) analysis, partial sequencing of the cytochrome c oxidase gene, and phenotypic examination via light microscopy.

### 2.2. Plasmids and Expression Constructs

In this study, SARS-CoV-2 spike (S) protein expression plasmids were constructed using the Gibson assembly method. Overlapping gene fragments (Thermo Fisher Scientific, Waltham, MA, USA) were combined with a linearized pCG1 plasmid, which had been digested with BamHI and XbaI. The assembly reaction was carried out using the Gibson Assembly HiFi Master Mix (Thermo Fisher Scientific, Waltham, MA, USA) and incubated at 56 °C for 1 h to facilitate fragment integration. Following assembly, the recombinant plasmids were introduced into Escherichia coli One Shot™ OmniMAX™ 2 T1R chemically competent cells (Thermo Fisher Scientific, Waltham, MA, USA) via transformation. Positive clones were identified through colony PCR screening, and their sequence accuracy was confirmed through Sanger sequencing, which was performed by a commercial sequencing service (Microsynth SeqLab, Göttingen, Germany). The expression plasmids employed for SARS-CoV-2 S protein production, as outlined below, included codon-optimized variants featuring a C-terminal truncation of the last 18 amino acids.

- pCG1-SARS-CoV-2 B.1 S∆18 (GISAID Accession ID: EPI_ISL_425259) [7];
- pCG1-SARS-CoV-2 JN.1 S∆18 (GISAID Accession ID: EPI_ISL_18530042) [20];
- pCG1-SARS-CoV-2 KP.3.1.1 S∆18 (GISAID Accession ID: EPI_ISL_19455032) [28];
- pCG1-SARS-CoV-2 XEC S∆18 (GISAID Accession ID: EPI_ISL_19454087) [28].

Constructs pCAGGS-DsRed, pCAGGS-VSV-G, pQCXIP-human ACE2-cMYC, pQCXIP-raccoon dog ACE2-cMYC, pQCXIP-pangolin ACE2-cMYC, pQCXIP-civet ACE2-cMYC, pQCXIP-cat ACE2-cMYC, pQCXIP-mouse ACE2-cMYC, pQCXIP-pig ACE2-cMYC, pQCXIP-Rhinolophus affinis ACE2-cMYC, pQCXIP-Rhinolophus sinicus ACE2-cMYC, pQCXIP-beta-galactosidase alpha fragment, and pQCXIP-beta-galactosidase omega fragment have been previously described [7,25,26]. The empty pCG1 expression plasmid was a gift from Roberto Cattaneo. Information on SARS-CoV-2 lineages and S protein sequences was retrieved from GISAID (https://gisaid.org/) and CoV-Spectrum (https://cov-spectrum.org/) databases. Access to these databases was made on 5 February 2025 (CoV-Spectrum).

### 2.3. Cell–Cell Fusion Assay

To assess S protein-driven cell–cell fusion, effector 293T cells were cotransfected with plasmids encoding S proteins or transfected with empty vectors jointly with a plasmid harboring the beta-galactosidase alpha fragment. Following transfection, the effector cells were washed, resuspended in the appropriate culture medium, and plated onto Calu-3-Omega target cells, which stably express the omega fragment of beta-galactosidase. In order to allow for S protein-driven cell–cell fusion, the co-culture was maintained for 18 h. Thereafter, a beta-galactosidase substrate (Gal-Screen, Thermo Fisher Scientific, Waltham, MA, USA) was added to the cells and the enzymatic reaction was permitted to proceed for 90 min. Subsequently, cell lysates were transferred into plates with white walls and a Hidex Sense plate luminometer (Hidex, Turku, Finland) was used to quantify luminescence.

### 2.4. Production of VSV Pseudoparticles (VSV_pp_) and Cell Entry Assays

Vesicular stomatitis virus pseudoparticles (VSV_pp_) were produced by transfecting 293T cells via calcium phosphate precipitation with plasmids encoding SARS-CoV-2 spike (S) protein variants, VSV-G (vesicular stomatitis virus glycoprotein, positive control), or DsRed (no viral protein, negative control). The cells were subsequently incubated for 1 h at 37 °C with 5% CO_2_ after being inoculated with a replication-deficient VSV that encodes for enhanced green fluorescent protein and firefly luciferase but lacks the genetic information for VSV-G (VSV*ΔG-Fluc, kindly provided by Gert Zimmer [29]). After removing the supernatant and washing with phosphate-buffered saline (PBS), cells (except those expressing VSV-G) were incubated with anti-VSV-G antibody-containing medium, while VSV-G-expressing cells received antibody-free medium. Following 16–18 h of incubation, the supernatant was centrifuged at 4000× *g* for 10 min to remove debris and was either used immediately for experimentation or stored at −80 °C. To assess pseudovirus entry efficiency, target cells were seeded into 96-well plates. BHK-21 target cells were transfected with plasmids encoding human ACE2, ACE2 orthologs from different animal species, or empty vector prior to pseudovirus transduction. For TMPRSS2-dependency studies, Calu-3 and Caco-2 cells were pretreated with camostat mesylate (Sigma Aldrich, St. Louis, MO, USA) or DMSO for 1 h at 37 °C. Equal volumes (100 µL/well) of pseudovirus were added, and cells were incubated at 37 °C with 5% CO_2_ for 16–18 h. Viral entry efficiency was determined by measuring firefly luciferase activity. Cells were lysed with PBS containing 0.5% Tergitol (Carl Roth, Karlsruhe, Germany) for 30 min, and luminescence was detected using Beetle-Juice substrate (PJK, Kleinblittersdorf, Germany) in a Hidex Sense plate luminometer (Hidex, Turku, Finland).

### 2.5. Immunoblot Analysis of S Protein Processing and Particle Incorporation

To analyze S protein particle incorporation and processing, particles containing SARS-CoV-2 S protein (or control particles without S. protein) were concentrated by centrifugation through a 20% (*w*/*v*) sucrose cushion in PBS. Samples were centrifuged at 25,000× *g* for 90 min at 4 °C. After centrifugation, particles pelleted through the sucrose cushion were mixed with an equal volume of 2× sample buffer (0.06 M Tris-HCl, 20% glycerol, 4% SDS, 5% β-mercaptoethanol, 0.4% bromophenol blue, 2 mM EDTA) and incubated at 96 °C for 15 min to ensure complete particle lysis. The lysates were separated by SDS-PAGE and proteins were transferred onto nitrocellulose membranes (Hartenstein, Würzburg, Germany). Membranes were blocked for 30 min at ambient temperature with PBS-T (0.05% Tween 20 in PBS; Carl Roth) supplemented with 5% bovine serum albumin (BSA; Carl Roth, Karlsruhe, Germany). For SARS-CoV-2 spike (S) protein detection, membranes were probed overnight at 4 °C with a rabbit anti-SARS-CoV-2 Spike S2 antibody (1:1000 dilution in PBS-T/5% BSA; SIN-40590-T62, Biozol, Hamburg, Germany). The VSV matrix protein (VSV-M) was included as a loading control and detected using a mouse anti-VSV-M [23H12] antibody (1:1000 in PBS-T/5% skim milk; EB0011, Kerafast, Boston, MA, USA). Following primary antibody incubation, membranes were washed three times with PBS-T (10 min per wash) and incubated for 1 h at room temperature with species-matched secondary antibodies: anti-rabbit IgG (H+L)-HRPO (1:2000 in PBS-T/5% skim milk; 111-035-003, Dianova, Eching, Germany) for S protein, and anti-mouse IgG (H+L)-HRPO (1:2000; 115-035-045, Dianova, Eching, Germany) for VSV-M. After three additional PBS-T washes, chemiluminescent signals were generated using a substrate solution (0.1 M Tris-HCl [pH 8.6], 250 µg/mL luminol, 0.1 mg/mL para-hydroxycoumaric acid, 1% H_2_O_2_) and imaged using a ChemoCam imaging system equipped with the ChemoStar Professional software (version v.0.3.23, Intas Science Imaging Instruments, Göttingen, Germany). ImageJ software (version 1.54d) was used to quantify the intensities of bands and calculate the intensity ratios.

### 2.6. Neutralization Assay

To evaluate the neutralizing capacity of monoclonal antibodies (mAbs), pseudovirus particles bearing the respective S proteins were prepared and incubated with varying concentrations of mAbs (ranging from 0.2 ng/mL to 2 µg/mL) at 37 °C for 30 min. After incubation, the mixtures were added to Vero cells and cultured for 16–18 h at 37 °C in a humidified 5% CO_2_ environment. Neutralization efficiency was assessed by quantifying the activity of the virus-encoded luciferase in cell lysates, which served as an indicator of S protein-mediated cell entry. Entry efficiency was normalized to control samples where pseudovirus particles were incubated without mAbs, which were set as 0% inhibition. The half-maximal inhibitory concentration (IC50) of mAbs was calculated using a non-linear regression model in GraphPad Prism (v6.07). mAbs were classified as neutralization-positive if their IC50 was ≤5 µg/mL, equivalent to 2.5 times the highest tested concentration.

### 2.7. Statistical Analysis and Quantification of Results

Data analysis was performed using Microsoft Excel (Office Professional Plus 2016) and GraphPad Prism (v6.07 or v8.4.3, GraphPad Software, Boston, MA, USA). Statistical significance was assessed with a two-tailed Student’s *t*-test with Welch correction and two-way ANOVA with Tukey’s multiple comparison test. The statistical test used for each experiment is specified in the figure legends. A *p*-value of ≤0.05 was considered significant (* *p* ≤ 0.05; ** *p* ≤ 0.01; *** *p* ≤ 0.001), while *p*-values > 0.05 were deemed not significant (ns).

## 3. Results

### 3.1. KP.3.1.1 and XEC S Proteins Are Efficiently Cleaved and Facilitate Robust Entry into Several Cell Lines

**Figure 1:**
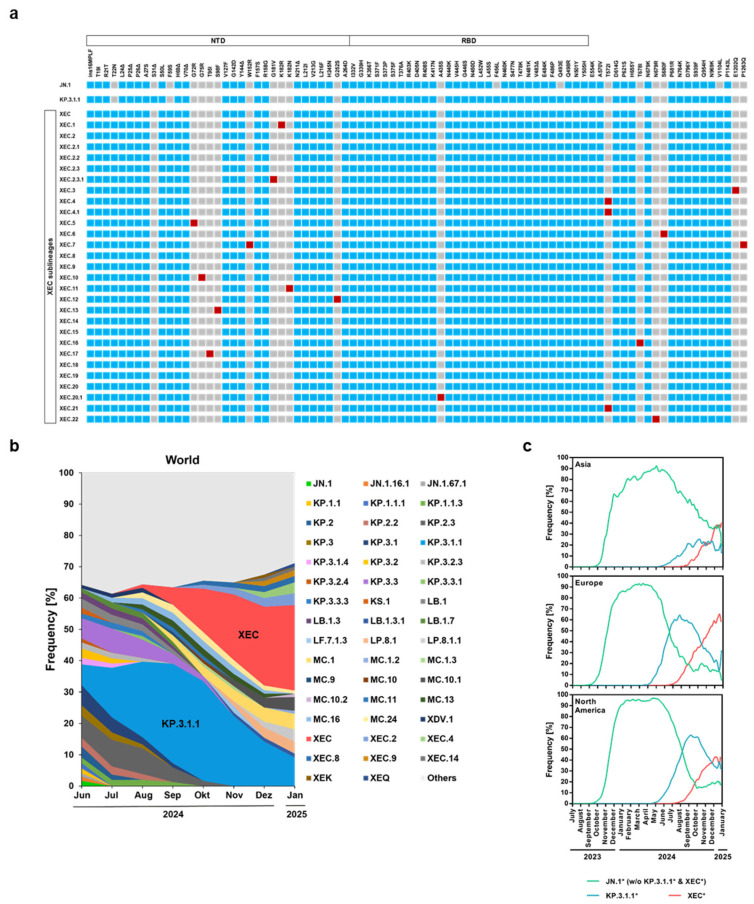
Spike protein mutations and global frequency of KP.3.1.1 and XEC lineages. (**a**) The mutations in the S proteins of various SARS-CoV-2 lineages (JN.1, KP.3.1.1, XEC, XEC.1, XEC.2, XEC.3, XEC.4, XEC.5, XEC.6, XEC.7, XEC.8, XEC.9, XEC.10, XEC.11, XEC.12, XEC.13, XEC.14, XEC.15, XEC.16, XEC.17, XEC.18, XEC.19, XEC.19, XEC.20, XEC.20.1, XEC.21, and XEC.22) are shown relative to the S protein of the Wuhan-01 isolate (color code: grey, no mutation at this position; blue, mutated residue; red, mutated residue that is acquired during XEC sublineage evolution). Abbreviations: N-terminal domain (NTD), receptor-binding domain (RBD). (**b**) The global frequencies of current SARS-CoV-2 lineages are shown and reflect the mean frequencies calculated using a sliding window of seven days. “Others” refers to lineages with frequencies of less than 1%. The information was obtained on 05.02.2025 from https://cov-spectrum.org/ (**c**) Frequency of SARS-CoV-2 lineages JN.1* (without KP.3.1.1* and XEC*) KP.3.1.1*, and XEC* in Asia, Europe, and North America. (Graphs are based on data obtained from https://cov-spectrum.org/).

The SARS-CoV-2 lineages KP.3.1.1 and XEC harbor distinct mutations in the S protein as compared to the ancestral JN.1 lineage (Figure 1a). The S protein of KP.3.1.1 is characterized by deletion S31del and mutations F456L, Q493E, and V1104L while the S protein of the XEC lineage is characterized by mutations T22N, F59S, F456L, Q493E, and V1104L, and XEC sublineages are diversifying (Figure 1a). Analysis of the frequency of detection of these variants from June 2024 to January 2025 showed that they have been continuously replacing JN.1 on the global level and are currently dominating (Figure 1b). The epidemiology showed the spread of JN.1, KP.3.1.1, and XEC in Europe, Asia, and North America (Figure 1c).

In order to analyze the host cell entry of KP.3.1.1 and XEC and its blockade by protease inhibitors and antibodies, we employed pseudovirus particles (_pp_), which adequately model SARS-CoV-2 entry and its inhibition [30,31]. Pseudovirus particles containing the respective S proteins, designated as KP.3.1.1_pp_ and XEC_pp_, were analyzed for S protein particle incorporation, cleavage, and cell entry efficiency. Pseudovirus particles bearing the JN.1 S protein (JN.1_pp_) served as the control.

**Figure 2:**
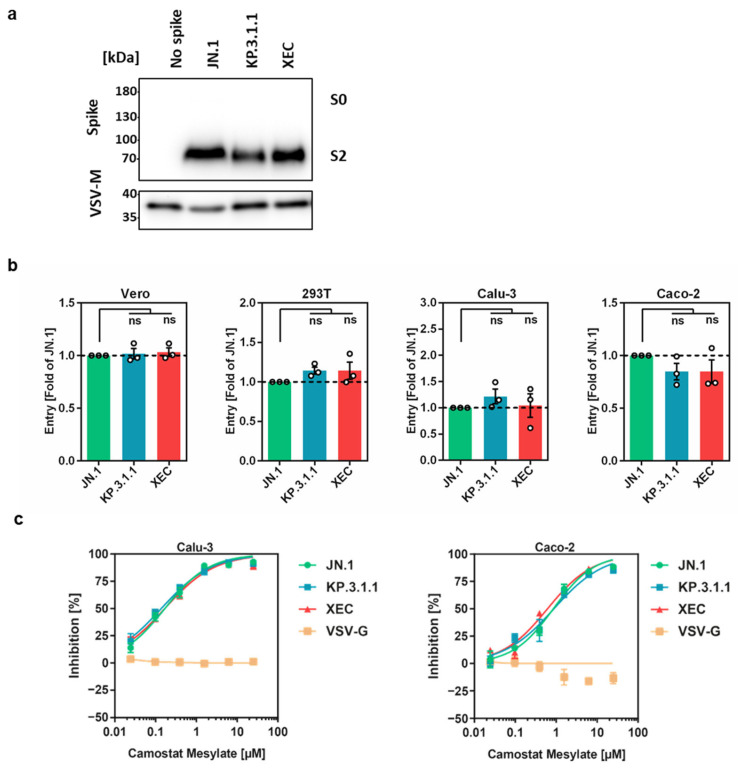
Spike protein cleavage, host cell entry, and protease dependency of pseudoviruses bearing KP.3.1.1 and XEC S proteins. (**a**) Spike protein particle incorporation and cleavage: Pseudovirus particles containing the indicated spike (S) proteins, along with a control lacking S protein, were concentrated via centrifugation. These particles were analyzed using SDS-PAGE followed by immunoblotting to assess S protein incorporation and cleavage. Vesicular stomatitis virus matrix protein (VSV-M) served as a loading control. Similar outcomes were observed across two independent experiments. (**b**) Host cell entry analysis: Pseudoviruses containing the indicated S proteins were inoculated onto the indicated cell lines. The efficiency of cell entry was assessed 16–18 h post-inoculation. Data represent the mean of three biological replicates, each comprising four technical replicates. Entry efficiency of pseudoviruses with the JN.1 S protein was used as the baseline (set at 1). Error bars denote the standard error of the mean (SEM). Additional details are provided in Supplementary Figure S1a. Statistical significance was evaluated using a two-tailed Student’s *t*-test with Welch correction (not significant [ns] *p* > 0.05). (**c**) Protease inhibition analysis: Pseudoviruses carrying the specified S proteins were inoculated onto Calu-3 and Caco-2 cells pretreated with the protease inhibitor camostat mesylate. Viral entry efficiency was quantified at 16–18 h after inoculation. Results reflect the average of three biological replicates, each performed with quadruplicate technical measurements. Cell entry efficiency in untreated controls (absence of inhibitor) set as 0%. Error bars represent the standard error of the mean (SEM). The original Western blot figures can be found in Supplementary Materials.

Immunoblotting demonstrated efficient particle incorporation and proteolytic cleavage of all S proteins (Figure 2a). Next, the efficiency of entry into the cell lines Vero (African green monkey, kidney), 293T (human, kidney), Caco-2 (human, colon), and Calu-3 (human, lung) was analyzed. Previous studies showed that SARS-CoV-2 entry into the TMPRSS2-negative cell lines Vero and 293T cells depends on cathepsin L activity [7] while entry into Caco-2 and Calu-3 cells largely depends on TMPRSS2 [7]. KP.3.1.1_pp_ and XEC_pp_ entered Vero, 293T, and Calu-3 cells with similar efficiency as JN.1_pp_ while entry into Caco-2 cells was slightly reduced (Figure 2b and Supplementary Figure S1a). Thus, the S proteins of KP.3.1.1 and XEC are robustly cleaved and mediate efficient entry into different cell lines.

### 3.2. Entry into Calu-3 and Caco-2 Cells Is TMPRSS2-Dependent

The reliance of JN.1 sublineages on TMPRSS2 for cell entry was evaluated using Calu-3 and Caco-2 cells, along with camostat mesylate, a serine protease inhibitor active against TMPRSS2 [25]. Treatment of cells with camostat mesylate effectively and dose-dependently inhibited entry of KP.3.1.1_pp_, XEC_pp_, and JN.1_pp_ into both Calu-3 and Caco-2 cells while VSV-G-driven entry was not inhibited (Figure 2c). These findings indicate that JN.1, KP.3.1.1, and XEC preserved the TMPRSS2 dependence reported for the ancestral variant, BA.2.86 [12,15].

### 3.3. Differential ACE2 Interactions of KP.3.1.1 and XEC S Proteins

**Figure 3:**
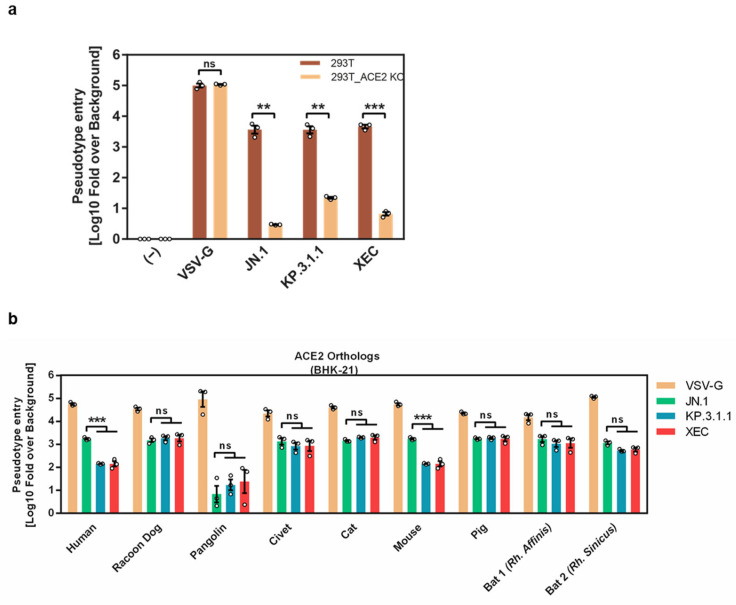
ACE2 usage by KP.3.1.1 and XEC. (**a**) Analysis of ACE2 dependence: Equal volumes of pseudoviruses harboring the specified S proteins were inoculated onto 293T and 293T-ACE2-KO cells. Cell entry was quantified at 16–18 h post-inoculation. The mean data from three biological replicates with four technical replicates each are presented. Entry levels were normalized against the background signal measured for particles lacking viral glycoproteins and log10 transformed. Error bars indicate the SEM. Statistical significance was determined using a two-tailed Student’s *t*-test with Welch correction (not significant [ns] *p* > 0.05; ** *p* ≤ 0.01; *** *p* ≤ 0.001). (**b**) Utilization of mammalian ACE2 orthologs: BHK-21 cells transiently expressing various mammalian ACE2 orthologs (or empty vector as a control) were infected with equal volumes of pseudoviruses carrying the indicated spike (S) proteins or VSV-G. The efficiency of S protein-mediated cell entry was determined by measuring firefly luciferase activity in cell lysates at 16–18 h post-inoculation. Data represent the mean of three biological replicates, each comprising four technical replicates, and are normalized against the background signal measured for particles lacking viral glycoproteins and log10 transformed. Error bars denote the SEM. Statistical significance was determined using a two-way ANOVA with Tukey’s multiple comparison test (not significant [ns] *p* > 0.05; *** *p* ≤ 0.001).

In order to determine whether JN.1, KP.3.1.1, and XEC depend on ACE2 for entry into human cells, entry into a 293T cell line was examined, in which ACE2 was knocked out via CRISPR/Cas9 [26,27]. ACE2 knock-out had no impact on the cell entry of control pseudotypes bearing the glycoprotein of vesicular stomatitis virus (VSV-G) but significantly reduced the entry of KP.3.1.1_pp_, XEC_pp_, and JN.1_pp_ close to background levels (Figure 3a). To initially characterize the interaction of KP.3.1.1 and XEC S proteins with the ACE2 receptor, we used BHK-21 cells transiently expressing human ACE2 or ACE2 orthologs from various species as mutations in the S protein can modulate S protein binding to animal ACE2 orthologs [32,33,34,35]. Directed expression of ACE2 had no impact on entry driven by VSV-G, as expected (Figure 3b). In contrast, S protein-driven entry into control BHK-21 cells was inefficient while directed expression of human ACE2 expression allowed for robust entry, although entry of JN.1_pp_ was more efficient than that measured for KP.3.1.1_pp_ and XEC_pp_ (Figure 3b). The JN.1 S protein efficiently utilized murine ACE2 while usage of this ACE2 ortholog by KP.3.1.1 and XEC S was significantly reduced (Figure 3b). Further, entry efficiency into cells expressing pangolin ACE2 was low across all S proteins tested (Figure 3b). Finally, the remaining ACE2 orthologs allowed for robust and comparable entry of all S protein-bearing pseudoviruses analyzed. In sum, KP.3.1.1_pp_ and XEC_pp_ depend on ACE2 for entry into human cells but, unlike JN.1_pp_, are unable to use murine ACE2 for robust cell entry.

### 3.4. Reduced Cell–Cell Fusion Mediated by KP.3.1.1 and XEC S Proteins Compared to JN.1 S Protein

**Figure 4:**
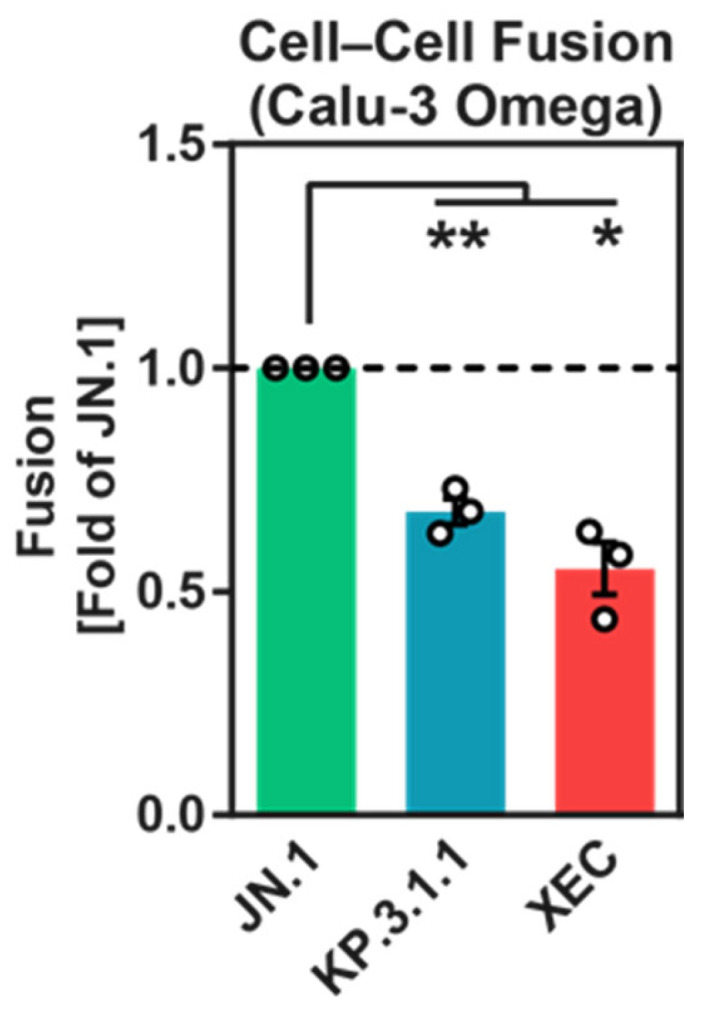
Cell–cell fusion mediated by KP.3.1.1 and XEC S proteins. 293T cells transiently transfected with plasmids encoding the specified spike (S) proteins or an empty vector (control) along with the beta-galactosidase alpha subunit were co-cultured with Calu-3-Omega cells stably expressing the beta-galactosidase omega subunit. At 18 h after cocultivation, a chemiluminescent beta-galactosidase substrate was added and luminescence was quantified. The graph presents normalized data derived from three biological replicates, each conducted with single samples. Fusion mediated by the JN.1 S protein was used as reference (set at 1). Additional details are provided in Supplementary Figure S1b. Error bars denote the SEM. Statistical significance was determined using a two-tailed Student’s *t*-test with Welch correction (* *p* ≤ 0.05; ** *p* ≤ 0.01). JN.1 vs. KP.3.1.1 *p* = 0.0081; JN.1 vs. XEC *p* = 0.0165.

Cell–cell fusion, a process facilitated by the SARS-CoV-2 S protein, is thought to contribute to disease pathogenesis [36,37,38]. To evaluate the ability of the KP.3.1.1 and XEC S proteins to drive cell–cell fusion, a split beta-galactosidase complementation assay was utilized. Effector cells (293T cells transfected with both S protein expression plasmids and the beta-galactosidase alpha-subunit) were co-cultured with target cells (Calu-3 cells engineered to stably express the beta-galactosidase omega-subunit). Fusion events were quantified by measuring beta-galactosidase activity in cell lysates. The results showed that cell–cell fusion mediated by KP.3.1.1 and XEC S proteins was significantly less efficient than fusion driven by the JN.1 S protein (Figure 4 and Supplementary Figure S1b). This indicates that mutations in the S proteins of KP.3.1.1 and XEC sublineages reduce their ability to fuse cells, potentially impacting their pathogenic potential.

### 3.5. KP.3.1.1_pp_ and XEC_pp_ Are Neutralized by Pre-Clinical Monoclonal Antibodies

**Figure 5:**
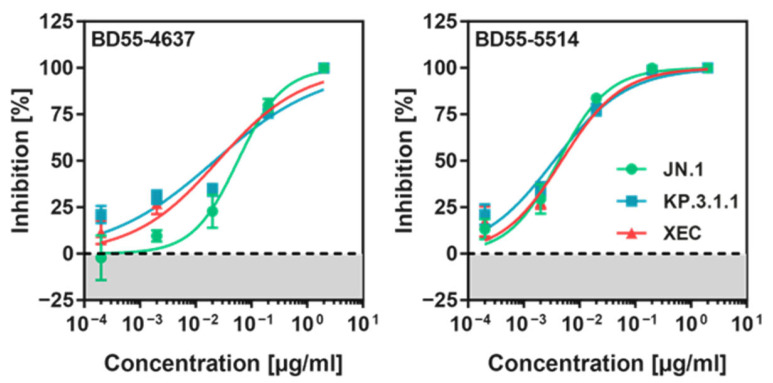
Neutralization of KP.3.1.1 and XEC by monoclonal antibodies. Equal volumes of pseudoviruses carrying the indicated spike (S) proteins were preincubated with various dilutions of the specified monoclonal antibodies (mAbs) before being inoculated onto Vero cells. Cell entry was quantified at 16–18 h post-inoculation. The graphs display the mean results from three biological replicates, each comprising four technical replicates. Entry levels in the absence of mAbs were used as the baseline for normalization (0% inhibition). Error bars denote the SEM.

Monoclonal antibodies (mAbs) have been pivotal in COVID-19 treatment and prevention [30]. However, SARS-CoV-2 variants have developed resistance to approved mAbs [12]. This study investigated the neutralizing activity of two pre-clinical mAbs, BD55-4637 and BD55-5514 (SA55), which were derived from vaccinated SARS-convalescent individuals [39] and have shown efficacy against the BA.2.86 variant [12]. To assess their effectiveness, KP.3.1.1_pp_, XEC_pp_, and JN.1_pp_ were preincubated with serial dilutions of each mAb and then inoculated onto Vero cells. Both BD55-4637 and BD55-5514 efficiently neutralized KP.3.1.1_pp_ and XEC_pp_ (Figure 5), with BD55-5514 maintaining robust activity across all tested S proteins while neutralization of JN.1_pp_ by BD55-4637 was slightly less efficient as compared to XEC_pp_ and KP.3.1.1_pp_.

## 4. Discussion

Our results show that the S proteins of KP.3.1.1 and XEC are proteolytically processed with high efficiency, mediate robust entry into several target cell lines, and rely on TMPRSS2 for Calu-3 lung cell entry. Further, entry driven by both S proteins was susceptible to inhibition by two pre-clinical monoclonal antibodies.

KP.3.1.1 and XEC pseudoviruses entered Vero, 293T, Calu-3, and Caco-2 cells with similar efficiency as JN.1_pp_, although Caco-2 cell entry was slightly reduced. The comparable entry into Vero cells is consistent with recent studies [28,40]. The present finding that JN.1_pp_, KP.3.1.1_pp,_ and XEC_pp_ enter Calu-3 lung cells with comparable efficiency diverges from our previous work showing reduced Calu-3 cell entry of KP.3.1.1_pp_ and XEC_pp_ as compared to JN.1_pp_ [28] and a preprint indicating increased Calu-3 cell entry of XEC_pp_ and, particularly, KP.3.1.1_pp_ relative to JN.1_pp_ [24]. These discrepancies likely relate to differences in the Calu-3 cell passage number, which may impact susceptibility to S protein-driven entry.

We and others previously reported that BA.2.86 is the first Omicron subvariant that enters Calu-3 lung cells with high efficiency and in a fully TMPRSS2-dependent manner [12,15]. The present study shows that TMPRSS2 usage was preserved upon evolution from BA.2.86 into JN.1 and, subsequently, the JN.1 sublineages KP.3.1.1 and XEC. Robust TMPRSS2 usage is believed to be a prerequisite to efficient viral spread in the lung and lung pathogenesis. Therefore, it will be interesting to examine the pathogenic potential of KP.3.1.1 and XEC in animal models. A preprint and a previous report revealed that JN.1 was attenuated in mice and hamsters regarding pathogenicity and transmission as compared to previous Omicron sublineages, indicating that presently unidentified attenuating mutations might outweigh the potentially augmented capacity for lung invasion conferred to JN.1 by S protein mutations [24,41].

A previous study reported that amino acid exchange L455S in JN.1 as compared to BA.2.86 S protein not only augments antibody evasion but also alters ACE2 interactions [41]. Our previous work showed that both KP.3.1.1 and XEC S protein bind to and employ human ACE2 with high efficiency for cell entry [28] and other studies reported similar findings [24,40]. Furthermore, we demonstrated that JN.1 as well as all JN.1 derivatives tested failed to employ pangolin ACE2 for robust entry while JN.1 but not KP.2.3, KP.3, and LB.1 were able to employ murine ACE2 for efficient cell entry [27]. Our present study confirms these findings and demonstrates that also KP.3.1.1 and XEC fail to efficiently use murine ACE2. This defect might preclude usage of WT mice for the analysis of KP.3.1.1 and XEC spread and pathogenesis.

We found that S proteins of KP.3.1.1 and XEC exhibit reduced cell–cell fusion, in keeping with previous studies [23,24], and the reduced cell–cell fusion activity of XEC S protein has been linked to mutation T22N, which introduces a N-glycosylation site into the XEC S protein [24]. Further, the pre-clinical antibodies BD55-4637 and BD55-5514 (SA55), which were obtained from COVID-19-vaccinated SARS patients, are directed against the RBD and show broad sarbecovirus-neutralizing activity [39,42], and neutralized JN.1_pp_, XEC_pp,_ and KP.3.1.1_pp_ with high efficiency. This finding is in keeping with the robust inhibition of JN.1_pp_ and KP.3_pp_ [27] by these antibodies and indicates that BD55-4637 and BD55-5514, jointly with other candidates [40], constitute potential therapeutic options for KP.3.1.1- or XEC-infected patients. The potential utility of these antibodies might extend to prophylaxis, particularly needed for high-risk populations.

Our findings reinforce the necessity of continued surveillance of emerging SARS-CoV-2 variants. The dynamic nature of viral evolution necessitates proactive monitoring to anticipate shifts in transmissibility, immune evasion, and pathogenic potential. Timely identification of key mutations, particularly those affecting TMPRSS2 dependence and antibody susceptibility, will be essential for guiding public health responses and ensuring the efficacy of both therapeutic and preventive interventions.

## 5. Conclusions

Our study provides evidence that the currently dominating SARS-CoV-2 variants KP.3.1.1 and XEC depend on TMPRSS2 activity for lung cell entry and exhibit reduced capacity to fuse target cells. Furthermore, we report that two monoclonal antibodies with broad activity against sarbecoviruses potently neutralize KP.3.1.1 and XEC. These findings emphasize the importance of ongoing surveillance of SARS-CoV-2 variants to preemptively address challenges posed by new mutations in the S protein.

## 6. Limitations of the Study

The pseudovirus model employed may not fully replicate all aspects of authentic SARS-CoV-2 infection. Further, infection studies were limited to cell lines and need to be extended to primary cells and/or organoids.

## Data Availability

Raw data are available upon request. This study did not generate code. All materials and reagents will be made available upon installment of a material transfer agreement.

## Supplementary Materials

The following supporting information can be downloaded at: https://www.mdpi.com/article/10.3390/vaccines13040385/s1, WB original figure; Figure S1: host cell entry and cell–cell fusion of the emerging JN.1 sublineages KP.3.1.1 and XEC.
