# Supplementary material for: Entry Efficiency, Protease Dependence, and Antibody-Mediated Neutralization of SARS-CoV-2 Sublineages KP.3.1.1 and XEC
Source: Vaccines (Basel). 2025 Apr 3;13(4):385. doi: 10.3390/vaccines13040385 (PMC12030816; doi:10.3390/vaccines13040385)
Supplement: Supplementary file 1 [file vaccines-13-00385-s001.zip › Figure S1.pdf]

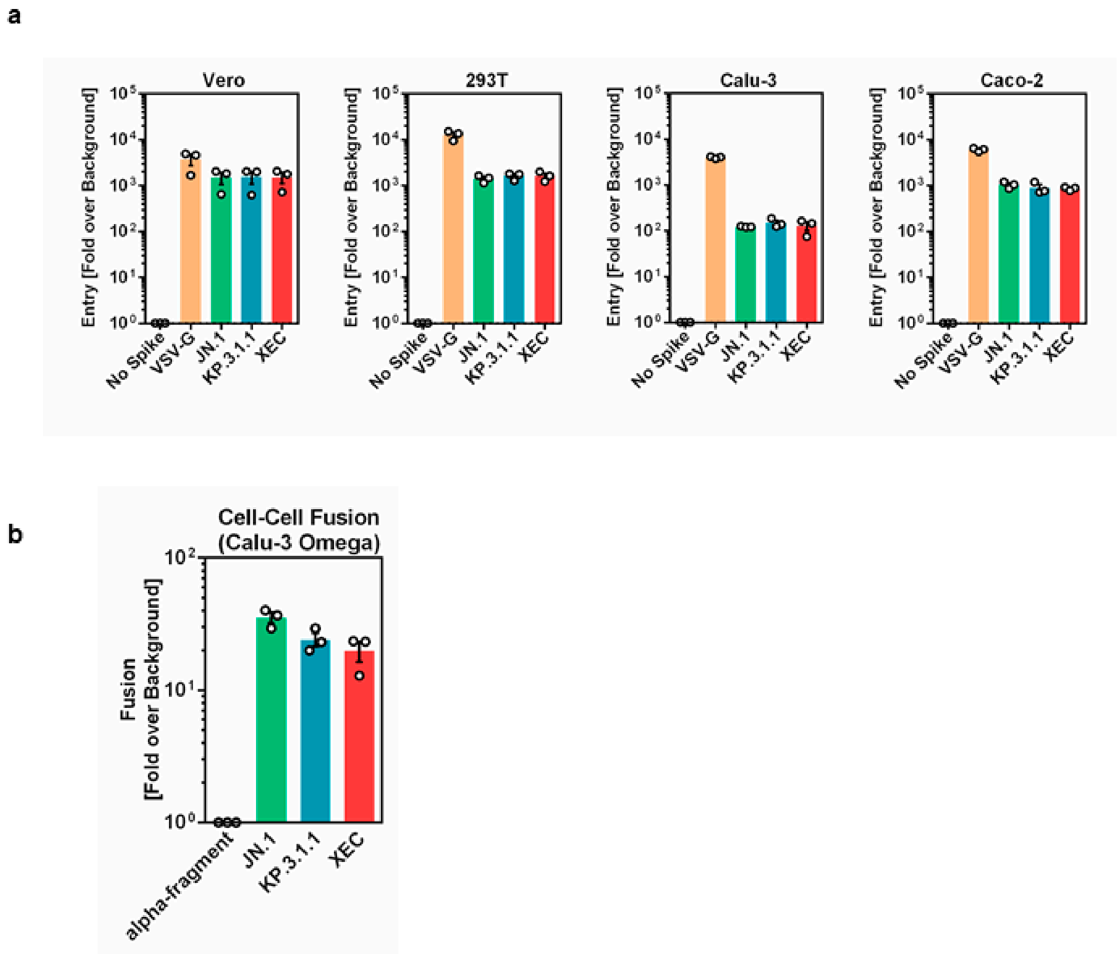

Figure S1. Host cell entry and cell-cell fusion of the emerging JN.1 sublineages KP.3.1.1 and XEC. (a) Host Cell Entry of SARS-CoV-2 Lineages: Pseudovirus particles containing the indicated spike (S) proteins, vesicular stomatitis virus glycoprotein (VSV-G; positive control), or no viral glycoprotein (negative control) were inoculated onto the specified cell lines. Entry efficiency was determined 16–18 hours post-inoculation by measuring firefly luciferase activity in cell lysates. Data represent the mean from three biological replicates, each conducted with four technical replicates. Entry levels were normalized to background signals from particles lacking viral glycoproteins (baseline set to 1). Error bars denote the SEM. (b) Cell-Cell Fusion: Effector 293T cells transfected to express the specified S proteins along with the beta-galactosidase alpha fragment were cocultured for 18 hours with target Calu-3 cells stably expressing the beta-galactosidase omega fragment. Cell-cell fusion mediated by S proteins was quantified by measuring reconstituted beta-galactosidase activity in the lysates. Data represent the mean from three biological replicates, each with three technical replicates, and were normalized to background signals from target cells coincubated with effector cells not expressing S proteins (baseline set to 1). Error bars denote the SEM.
