# Supplementary material for: Entry Efficiency, Protease Dependence, and Antibody-Mediated Neutralization of SARS-CoV-2 Sublineages KP.3.1.1 and XEC
Source: Vaccines (Basel). 2025 Apr 3;13(4):385. doi: 10.3390/vaccines13040385 (PMC12030816; doi:10.3390/vaccines13040385)
Supplement: Supplementary file 1 [file vaccines-13-00385-s001.zip › WB original figure.pptx]

## Slide 1
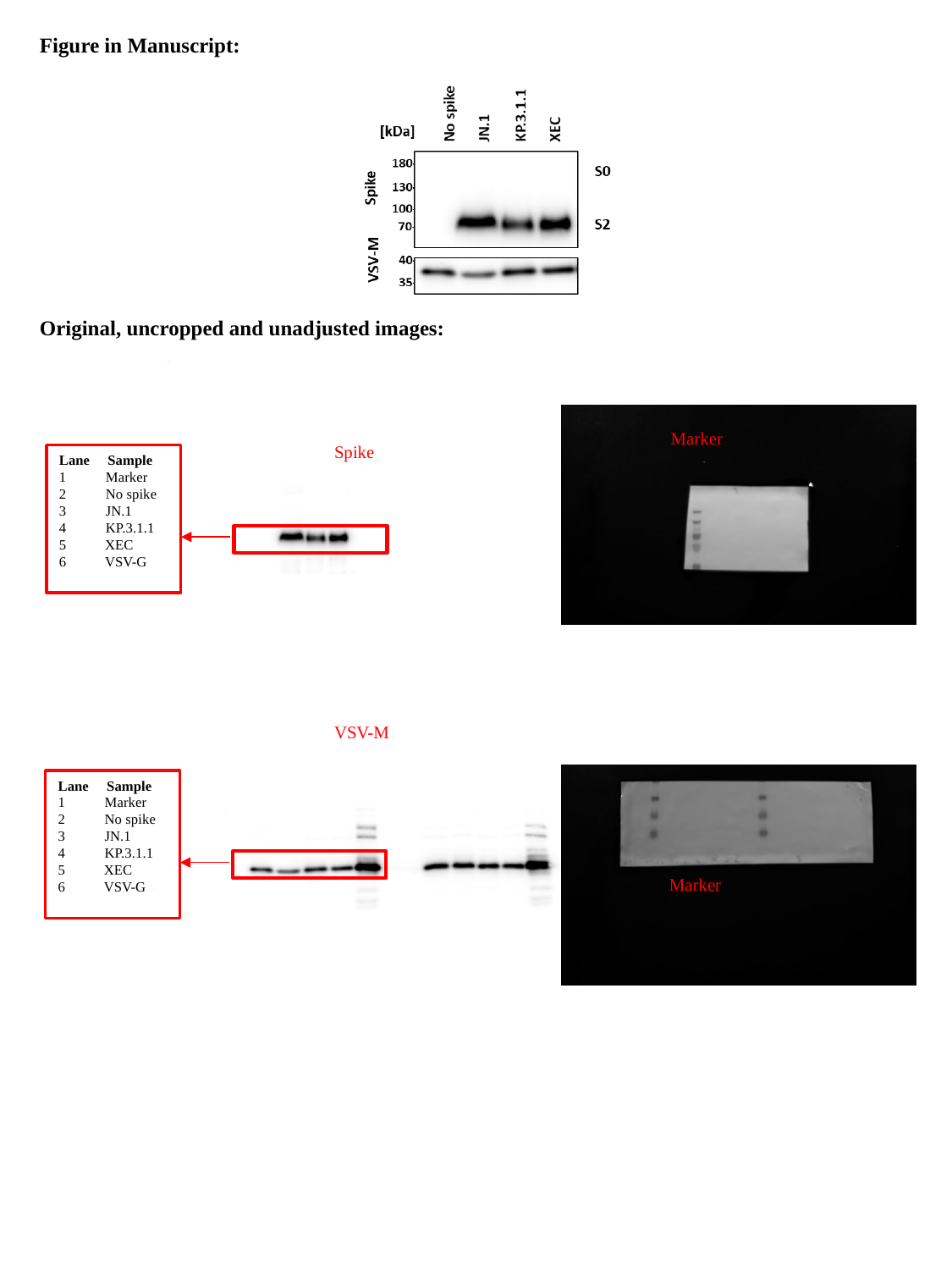

Figure in Manuscript:
Original, uncropped and unadjusted images:
Marker
Spike
Lane Sample
1 Marker
2 No spike
3 JN.1
4 KP.3.1.1
 XEC
 VSV-G
VSV-M
Lane Sample
1 Marker
2 No spike
3 JN.1
4 KP.3.1.1
 XEC
 VSV-G
Marker

## Slide 2
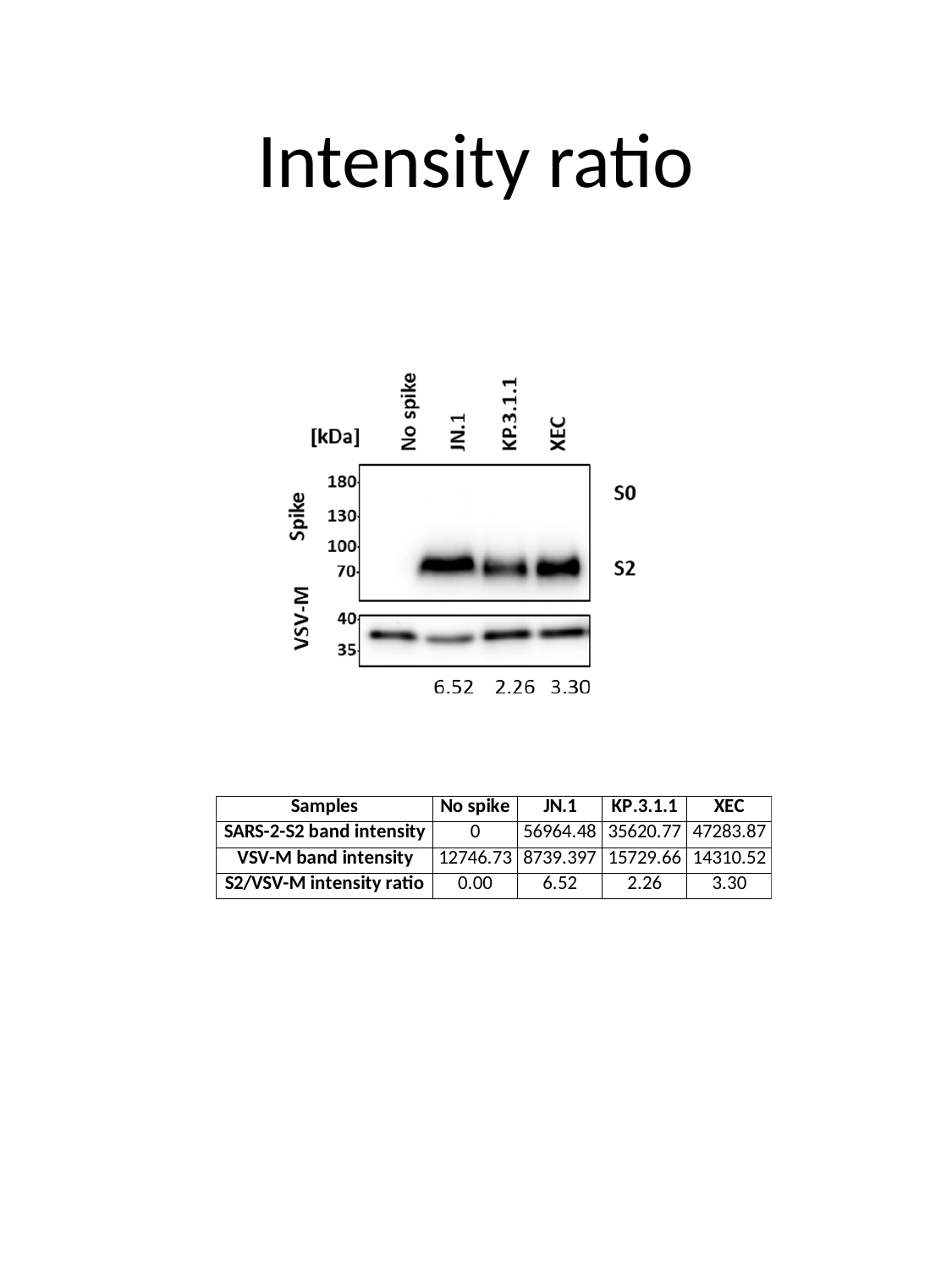

# Intensity ratio
